# Incidence of ischaemic heart disease and stroke among people with psychiatric disorders: retrospective cohort study

**DOI:** 10.1192/bjp.2019.250

**Published:** 2020-08

**Authors:** Caroline A. Jackson, Joannes Kerssens, Kelly Fleetwood, Daniel J. Smith, Stewart W. Mercer, Sarah H. Wild

**Affiliations:** 1 Chancellor's Fellow, Usher Institute of Population Health Sciences and Informatics, University of Edinburgh, Scotland, UK; 2 Principal Information Analyst, Information Services Division, National Services Scotland, NHS Scotland, Scotland, UK; 3 Statistician, Usher Institute of Population Health Sciences and Informatics, University of Edinburgh, Scotland, UK; 4 Professor, Institute of Health and Wellbeing, University of Glasgow, Scotland, UK; 5 Professor, Usher Institute of Population Health Sciences and Informatics, University of Edinburgh, Scotland, UK

**Keywords:** Stroke, schizophrenia, bipolar disorder, depression, ischaemic heart disease

## Abstract

**Background:**

Psychiatric disorders are associated with increased risk of ischaemic heart disease (IHD) and stroke, but it is not known whether the associations or the role of sociodemographic factors have changed over time.

**Aims:**

To investigate the association between psychiatric disorders and IHD and stroke, by time period and sociodemographic factors.

**Method:**

We used Scottish population-based records from 1991 to 2015 to create retrospective cohorts with a hospital record for psychiatric disorders of interest (schizophrenia, bipolar disorder or depression) or no record of hospital admission for mental illness. We estimated incidence and relative risks of IHD and stroke in people with versus without psychiatric disorders by calendar year, age, gender and area-based deprivation level.

**Results:**

In all cohorts, incidence of IHD (645 393 events) and stroke (276 073 events) decreased over time, but relative risks decreased for depression only. In 2015, at the mean age at event onset, relative risks were 2- to 2.5-fold higher in people with versus without a psychiatric disorder. Age at incidence of outcome differed by cohort, gender and socioeconomic status. Relative but not absolute risks were generally higher in women than men. Increasing deprivation conveys a greater absolute risk of IHD for people with bipolar disorder or depression.

**Conclusions:**

Despite declines in absolute rates of IHD and stroke, relative risks remain high in those with versus without psychiatric disorders. Cardiovascular disease monitoring and prevention approaches may need to be tailored by psychiatric disorder and cardiovascular outcome, and be targeted, for example, by age and deprivation level.

## Background

An estimated 8 million deaths (14.3% of all deaths) annually worldwide are attributable to mental disorders.^1^ Among people with psychiatric disorders (defined here as schizophrenia, bipolar disorder and major depression), life expectancy is reduced by up to 20 years compared with the general population.^2–5^ Although the risk of death from unnatural causes, such as suicide, is markedly increased in people with psychiatric disorders, premature mortality is largely owing to natural causes of death, including cardiovascular and cerebrovascular disease (collectively termed CVD hereafter).^1,6–8^ Although estimates vary across studies, by psychiatric disorder and specific outcomes, having a psychiatric disorder is associated with about a two-fold increased risk of CVD occurrence.^9–11^ However, this is largely based on studies of psychiatric disorders and CVD mortality. Relatively few studies have examined CVD incidence and these were generally small in terms of numbers of CVD outcomes, especially among those with schizophrenia and bipolar disorder.^10,11^ Furthermore, the evidence for an association between bipolar disorder and ischaemic heart disease (IHD) remains mixed and, when meta-analysed, inconclusive.^9,11^

## Justification for study

To our knowledge, no study has reported time trends in CVD incidence by psychiatric disorder, and so it is unclear whether the difference in risk of CVD has narrowed or widened over time. Furthermore, previous studies have rarely explored whether the association between psychiatric disorder and CVD occurrence differs by gender. Having a psychiatric disorder has been linked to a greater risk of downward social mobility,^12^ and the excess mortality associated with these disorders is more marked in the lowest socioeconomic groups.^12^ However, the relationship between psychiatric disorder, socioeconomic status and incidence of chronic physical diseases such as IHD and stroke, remains unexplored in longitudinal studies. Determining how the effect of psychiatric disorders on IHD and stroke risk varies by age, gender and socioeconomic status might deepen our understanding of the relationship between psychiatric disorders and IHD and stroke risk, and raise implications for cardiovascular disease prevention.

We therefore conducted a cohort study to determine the incidence of IHD and stroke among people admitted to hospital with schizophrenia, bipolar disorder or depression, compared with the Scottish population without a record of hospital admission for any mental disorder, and to examine whether these associations differed by age, gender, area-based deprivation or time period.

## Method

This study was reported in accordance with the Standard Reporting of Observational Studies in Epidemiology (STROBE)^13^ and Reporting of Studies Conducted using Observational Routinely-Collected Health Data (RECORD)^14^ statements.

Permission to conduct this study with pseudonymised, non-consented data was obtained from the Public Benefit and Privacy Panel of National Health Service National Services Scotland, reference number 1516-0626.

### Data sources

In Scotland, routine administrative health data-sets are linkable deterministically, via an individual community health index number. Area-based deprivation is measured by the Carstairs Index and the Scottish Index of Multiple Deprivation. In this study we used the Carstairs Index,^15^ which is recommended in Scotland as the most appropriate deprivation measure to use for long-term time-trend analyses that include pre-1996 data.^16^ The Carstairs Index is a relative measure of material deprivation, comprising an unweighted combination of four standardised census variables: lack of car ownership, low occupational social class, overcrowded households and male unemployment. Carstairs Index scores are obtained by summing together the standardised values of the four components to create deprivation scores for each postcode sector (containing an average of about 5000 people).^15^ We identified Carstairs Index scores from the record of IHD and stroke events resulting in hospital admission or death.

We identified IHD and stroke events requiring admission to hospital occurring between 1 January 1991 and 31 December 2015 from the General Acute Inpatient and Day Case – Scottish Morbidity Records (SMR01, see https://www.ndc.scot.nhs.uk/Data-Dictionary/SMR-Datasets/SMR01-General-Acute-Inpatient-and-Day-Case/), and fatal out-of-hospital IHD and stroke events from the National Records of Scotland Death Records (see https://www.ndc.scot.nhs.uk/national-datasets/data.asp?SubID=13). SMR01 includes details of all acute hospital admissions in Scotland routinely from 1980 onward. We defined IHD events requiring hospital admission by ICD-10 (see https://icd.who.int/browse10/2016/en#XX) codes I20–I25 and ICD-9 codes 410–414, and strokes requiring hospital admission by ICD-10 codes I60, I61, I63 and I64 and ICD-9 codes 430, 431, 433, 434 and 436 (where codes were included in either a primary or secondary diagnosis field). We ascertained deaths due to IHD and stroke where these were attributed as the primary cause of death, using the same ICD-10 and ICD-9 codes as above. We defined IHD and stroke events occurring at age ≥18 years as incident if these were the first to occur between 1 January 1991 and 31 December 2015, where no previous admissions to hospital for IHD or stroke had been recorded during the preceding 10 years (i.e. a 10-year look-back was applied, consistent with the definition of incident IHD and stroke events in data published by the National Health Service Scotland Information Services Division). Because of the low numbers of events at younger ages, we restricted analyses to those aged ≥40 years.

We used the Scottish Mental Health Inpatient and Day Case (SMR04) data-set (which records all psychiatric hospital admissions from 1981 onward) and SMR01 to identify people with a hospital record for any mental illness listed in the mental and behavioural disorders chapters for ICD-9 and ICD-10 (except for organic disorders (codes F00–F09) and mental retardation (codes F70–F79)). We identified people with a history of admission to hospital for schizophrenia (ICD-9 codes 295.0–295.3 and 295.6–295.9, and ICD-10 codes F20 and F25), bipolar disorder (ICD-9 codes 296.0–296.1 and 296.4–296.7, and ICD-10 codes F30–F31) or depression (ICD-9 codes 296.2–296.3, 298.0 and 311, and ICD-10 codes F32 and F33) where the hospital admission occurred among adults aged ≥18 years (Supplementary Table 1 available at https://doi.org/10.1192/bjp.2019.250). We classified schizoaffective disorders as ‘schizophrenia’ because it comes under the ICD-10 classification subheading of ‘Schizophrenia, schizotypal and delusional disorders’. We applied a hierarchy of severity to assign people to one group only, ranking disorders in decreasing severity as schizophrenia, bipolar disorder and depression.

### Statistical analyses

We conducted analyses for IHD and stroke separately, including people aged ≥40 years. Our study cohorts comprised people with a record of schizophrenia, bipolar disorder or depression, and a comparison group of people without a hospital record of any mental illness listed in the mental and behavioural disorders chapters for ICD-9 and ICD-10 (except for organic disorders and mental retardation). Using the previously described hospital admission and death data, we summed incident IHD and stroke events by the stratifying variables of calendar year, age, gender and deprivation quintile for each of our three psychiatric disorder groups. To calculate the IHD and stroke event numbers by each of these stratifying variables for the comparison group without any mental illness, we subtracted the number of incident IHD or stroke events occurring among those with an admission to hospital for any mental illness from the total number of IHD or stroke hospital admission events for the general population.

Similarly, we used hospital admission and death data to estimate person-time-at-risk for the three psychiatric disorder groups. Each individual's person-time was estimated as the number of days from the start of the study period (1 January 1991), or date of admission to hospital for schizophrenia, bipolar disorder or depression if this occurred during the study period, to the date of admission to hospital for IHD or stroke, death or end of study period (31 December 2015). These were summed by calendar year, age, gender and deprivation quintile. We obtained mid-year total population estimates by age, gender and quintile of area-based deprivation from the National Records of Scotland. For the comparison group of no history of admission to hospital for any mental illness, we estimated person-years-at-risk by subtracting the person-time for those with a hospital admission for mental illness from these mid-year total population estimates.^15^

We calculated age-standardised IHD and stroke incidence rates, per 1000 person-years, using the 2013 European Standard Population (see http://www.isdscotland.org/Products-and-Services/GPD-Support/Population/Standard-Populations/ for more details). We modelled incidence with quasi-Poisson models. Quasi-Poisson models were required to allow for overdispersion. We fitted separate models for IHD and stroke incidence. The models included main effects for history of a psychiatric disorder, age, gender, time period and area-based deprivation index and all two-way interactions with each of history of psychiatric disorder and age. Age (using the mid-point of each 5-year block) and time period were modelled as continuous variables, using natural cubic splines. Area-based deprivation index was modelled as a categorical variables. The models were used to calculate relative risks. Here, we illustrate our general findings by presenting data in figures for the most recent year available (2015), the middle deprivation quintile, and 65 years of age for IHD and 70 years of age for stroke (these were the 5-year block mid-points that were closest to the mean age at which IHD and stroke occurred across the whole population combined). Interactive versions of each figure, where results for all ages, years and deprivation groups are shown, are available online (see https://uiphsi-mdcvddm.shinyapps.io/cvd-inc-mmd/). All analyses were conducted with R software for Windows, version 3.3.3.^17^

## Results

### Descriptive characteristics

Between 1990 and 2015, we identified 645 393 IHD and 276 073 stroke events among people with no history of admission to hospital for any mental illness. Among people with schizophrenia, bipolar disorder and depression, there were 3180, 4927 and 11 260 IHD events, respectively, and 1433, 2600 and 5668 stroke events, respectively. IHD and strokes occurred at a younger age in those with schizophrenia (mean, 63.1 years ± 13.1 s.d. and 66.8 years ± 13.8 s.d., respectively) compared with the group without a hospital admission for mental illness (mean, 71.1 ± 12.8 s.d. and 74.4 ± 13.4 s.d., respectively). Mean age at IHD or stroke event was also lower in those with a hospital record for depression, but similar among those with bipolar disorder (Table 1).

**Table 1:** Characteristics of all people aged ≥40 years with an IHD or stroke event, by hospital admission for psychiatric disorder, in Scotland 1991–2015

Characteristic	No mental illness	Schizophrenia	Bipolar disorder	Depression
IHD, *n*	626 026	3180	4927	11 260
Male, *n* (%)	338 322 (54.0)	1741 (54.8)	1831 (37.2)	4701 (41.8)
Mean age at IHD event, years (±s.d.)	71.1 (±12.8)	63.1 (±13.1)	70.0 (±12.4)	67.6 (±14.6)
All [men:women]	[68.4 ± 12.5:74.2 ± 12.4]	[59.6 ± 12.7:67.3 ± 12.4]	[67.8 ± 12.3:71.3 ± 12.2]	[64.8 ± 14.1:69.6 ± 14.6]
Area-based deprivation^a^ quintile				
1, most deprived	140 959 (22.5)	1012 (31.8)	1261 (25.6)	3029 (26.9)
2	130 237 (20.8)	732 (23.0)	1056 (21.4)	2535 (22.5)
3	126 208 (20.2)	613 (19.3)	924 (18.8)	2263 (20.1)
4	122 025 (19.5)	463 (14.6)	916 (18.6)	1932 (17.2)
5, least deprived	106 597 (17.0)	360 (11.3)	770 (15.6)	1501 (13.3)
Stroke, *n*	266 372	1433	2600	5668
Male, *n* (%)	116 840 (43.9)	646 (45.1)	772 (29.7)	2074 (36.6)
Mean age at stroke, years (±s.d.)	74.4 (±13.4)	66.8 (±13.8)	74.0 (±12.1)	72.7 (±14.1)
All [men:women]	[71.4 ± 13.0:76.8 ± 13.2]	[62.5 ± 13.7:70.3 ± 12.8]	[70.9 ± 11.5:75.3 ± 12.1]	[69.8 ± 13.7:74.4 ± 14.4]
Area-based deprivation^a^ quintile				
1, most deprived	57 761 (21.7)	429 (29.9)	589 (22.7)	1337 (23.6)
2	53 948 (20.3)	307 (21.4)	508 (19.5)	1228 (21.7)
3	53 170 (20.0)	276 (19.3)	517 (19.9)	1143 (20.2)
4	53 736 (20.2)	211 (14.7)	533 (20.5)	1021 (18.0)
5, least deprived	47 757 (17.9)	210 (14.7)	453 (17.4)	939 (16.6)

IHD, ischaemic heart disease.

a. Based on Carstairs Index.

In all groups, mean age at IHD and stroke incidence was considerably lower in men than women, and was inversely associated with deprivation (Supplementary Figure 1). People with a psychiatric disorder and an IHD or stroke event were more likely to be in the most deprived quintile than those without previous hospital admission for any mental illness (Table 1).

Over time, age-standardised rates of IHD and stroke decreased in all groups, but remained markedly higher among men and women with a hospital record for a psychiatric disorder compared with people without prior hospital admission for any mental illness (Supplementary Table 2).

### Mental health status and IHD and stroke rates by age

As expected, IHD and stroke incidence generally increased with increasing age among all groups, as illustrated by estimates for 2015 shown in Supplementary Figure 1. Up to about 80 years of age the incidence was higher in those with a hospital record for schizophrenia and bipolar disorder than in those with no hospital admission for mental illness. IHD and stroke incidence remained higher up to about 95 years of age in those with a hospital record for depression compared with people with no hospital record of mental illness.

### Mental health status and IHD and stroke rates by area-based deprivation

In general, IHD and stroke incidence among people with no mental illness were positively associated with deprivation level. We observed similar patterns among people with bipolar disorder and depression, but a slightly less steep gradient among those with schizophrenia (Supplementary Figure 2). Thus, the relative difference in IHD risk between the schizophrenia and no mental illness groups was slightly lower in the most deprived versus the least deprived group in both men (in 2015: relative risk 1.57, 95% CI 1.34–1.84 and relative risk 1.81, 95% CI 1.49–2.20, respectively) and women (in 2015: relative risk 2.06, 95% CI 1.74–2.43 and relative risk 2.36, 95% CI 1.94–2.88, respectively; Table 2), but the absolute difference in IHD risk was generally similar for the most and least deprived quintiles (Supplementary Figure 3). In contrast, stroke risk in those with schizophrenia varied little by deprivation level (Supplementary Figure 2), with larger absolute and relative differences (when compared with the no mental illness group) in the least deprived versus the most deprived group, in men (in 2015: relative risk 1.51, 95% CI 1.21–1.88 and relative risk 2.05, 95% CI 1.60–2.64, respectively) and women (in 2015: relative risk 1.82, 95% CI 1.46–2.27 and relative risk 2.48, 95% CI 1.93–3.18, respectively; Table 2 and Supplementary Figure 2). Relative risks for IHD and stroke comparing bipolar disorder and depression with no mental illness were broadly similar across deprivation groups, with the clearest differences in relative risks seen between the most and least deprived groups (Table 2). Of particular importance is that absolute differences in IHD (but not stroke) risk were greater in the most deprived versus the least deprived group when comparing bipolar disorder and depression with the no mental illness group (Supplementary Figure 3).

**Table 2:** Relative risks for ischaemic heart disease and stroke, among people aged 65 and 70 years, comparing people with a hospital admission record for each mental disorder versus no mental illness, by gender and area-based deprivation quintile, in Scotland in 2010 and 2015, based on predictive values from quasi-Poisson regression models

Year	Deprivation quintile	Men			Women		
		Relative risk (95% CI)			Relative risk (95% CI)		
		Schizophrenia	Bipolar disorder	Depression	Schizophrenia	Bipolar disorder	Depression
Ischaemic heart disease							
2010	1 (most deprived)	1.40 (1.25–1.58)	1.61 (1.45–1.79)	1.89 (1.77–2.01)	1.83 (1.62–2.07)	1.90 (1.72–2.10)	2.42 (2.27–2.58)
	2	1.56 (1.38–1.77)	1.61 (1.44–1.80)	2.20 (2.06–2.36)	2.04 (1.79–2.33)	1.90 (1.71–2.11)	2.83 (2.65–3.03)
	3	1.71 (1.50–1.95)	1.57 (1.40–1.75)	2.25 (2.10–2.41)	2.23 (1.95–2.56)	1.85 (1.66–2.07)	2.89 (2.69–3.10)
	4	1.70 (1.46–1.96)	1.63 (1.45–1.83)	2.18 (2.03–2.35)	2.22 (1.91–2.58)	1.92 (1.72–2.15)	2.80 (2.60–3.02)
	5 (least deprived)	1.61 (1.37–1.90)	1.42 (1.26–1.60)	2.08 (1.91–2.26)	2.11 (1.79–2.48)	1.68 (1.49–1.89)	2.67 (2.46–2.90)
2015	1 (most deprived)	1.57 (1.34–1.84)	1.64 (1.41–1.91)	2.00 (1.84–2.18)	2.06 (1.74–2.43)	1.94 (1.67–2.26)	2.57 (2.36–2.80)
	2	1.75 (1.48–2.07)	1.64 (1.40–1.92)	2.34 (2.14–2.55)	2.29 (1.93–2.72)	1.94 (1.66–2.26)	3.00 (2.75–3.28)
	3	1.91 (1.61–2.27)	1.60 (1.37–1.87)	2.39 (2.18–2.61)	2.50 (2.10–2.98)	2.50 (2.10–2.98)	3.07 (2.80–3.35)
	4	1.90 (1.58–2.28)	1.66 (1.42–1.95)	2.32 (2.11–2.54)	2.49 (2.06–3.00)	1.96 (1.68–2.30)	2.98 (2.71–3.26)
	5 (least deprived)	1.81 (1.49–2.20)	1.45 (1.23–1.71)	2.20 (2.00–2.43)	2.36 (1.94–2.88)	1.71 (1.46–2.02)	2.83 (2.56–3.12)
Stroke							
2010	1 (most deprived)	1.67 (1.42–1.96)	1.66 (1.44–1.91)	2.30 (2.11–2.51)	2.01 (1.71–2.37)	1.82 (1.60–2.07)	2.30 (2.11–2.50)
	2	1.80 (1.51–2.14)	1.68 (1.45–1.94)	2.78 (2.55–3.04)	2.17 (1.82–2.59)	1.84 (1.61–2.11)	2.78 (2.55–3.04)
	3	2.07 (1.73–2.48)	1.93 (1.68–2.23)	2.84 (2.59–3.11)	2.50 (2.09–2.98)	2.12 (1.86–2.42)	2.84 (2.60–3.11)
	4	1.95 (1.60–2.38)	1.91 (1.66–2.20)	2.69 (2.45–2.96)	2.35 (1.94–2.86)	2.10 (1.83–2.40)	2.70 (2.46–2.96)
	5 (least deprived)	2.27 (1.86–2.77)	1.66 (1.44–1.93)	2.91 (2.64–3.21)	2.75 (2.26–3.34)	1.83 (1.58–2.11)	2.92 (2.65–3.21)
2015	1 (most deprived)	1.51 (1.21–1.88)	1.54 (1.27–1.87)	2.10 (1.88–2.35)	1.82 (1.46–2.27)	1.69 (1.40–2.04)	2.10 (1.88–2.35)
	2	1.62 (1.29–2.05)	1.56 (1.28–1.90)	2.54 (2.27–2.85)	1.96 (1.55–2.48)	1.71 (1.41–2.08)	2.55 (2.28–2.85)
	3	1.87 (1.48–2.37)	1.80 (1.48–2.18)	2.60 (2.32–2.91)	2.26 (1.79–2.86)	1.97 (1.63–2.39)	2.60 (2.32–2.91)
	4	1.76 (1.37–2.26)	1.78 (1.46–2.16)	2.46 (2.19–2.77)	2.13 (1.66–2.73)	1.95 (1.61–2.37)	2.47 (2.19–2.77)
	5 (least deprived)	2.05 (1.60–2.64)	1.55 (1.27–1.89)	2.66 (2.36–3.00)	2.48 (1.93–3.18)	1.70 (1.39–2.07)	2.67 (2.37–3.01)

### Mental health status and IHD and stroke incidence by gender

The incidence of IHD and stroke was higher among men than women in all cohorts. Although relative risks associated with psychiatric disorders were larger for women than men for both IHD and stroke in all deprivation quintiles and for all three psychiatric disorders (Table 2), confidence intervals between genders overlapped. Also, the absolute differences in risk of IHD and stroke between comparison groups were generally similar for men and women (Supplementary Figure 3).

### Mental health status and IHD and stroke incidence over time

**Fig. 1:**
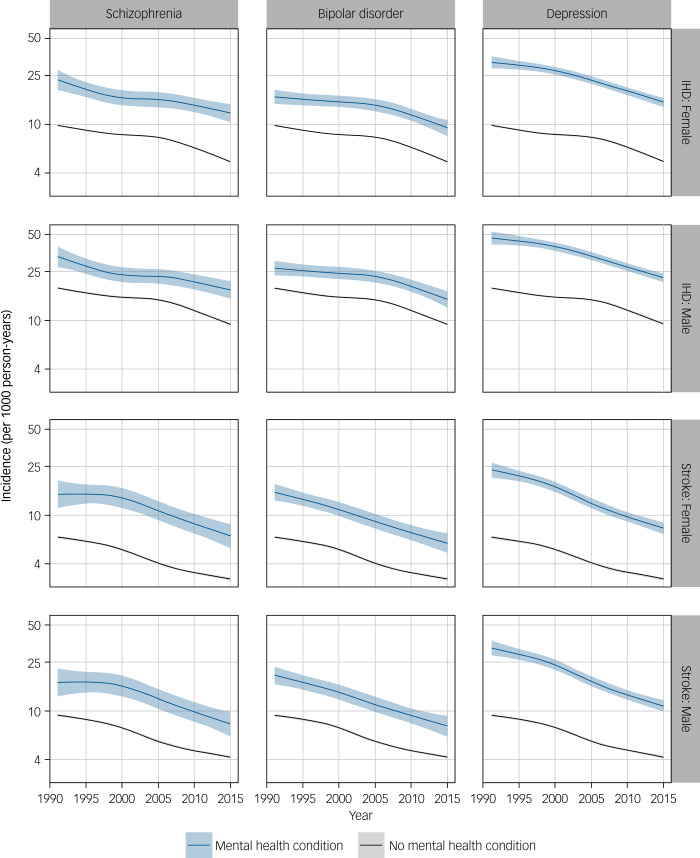
Incidence (per 1000 person-years) of ischaemic heart disease (IHD) and stroke in people in the middle deprivation quintile and aged 65 (IHD) and 70 (stroke) years, for each mental health condition and by gender, in Scotland, from 1991 to 2015. Predicted incidence rates obtained from quasi-Poisson regression models including mental health status, age, gender, deprivation and time period, plus all mental health status and age interactions.

There was a decline in IHD and stroke rates over time in all comparison groups (data for 65-year-olds illustrated in Fig. 1). We observed similar time patterns for all deprivation categories and ages, apart from those aged 40–49 years, where there was little change in IHD and stroke incidence over time. Among people with schizophrenia and bipolar disorder, relative risks of IHD and stroke risk remained fairly constant over time (Supplementary Figure 4). In 2015, schizophrenia was associated with an almost two-fold increased risk of IHD and stroke in men and a more than two-fold increased risk in women (Table 3). The relative effect of bipolar disorder on IHD was slightly lower than that of schizophrenia. The difference in absolute risk of IHD among those with depression versus no psychiatric illness decreased slightly over time, but the relative effect remained stable, with depression associated with an almost 2.5-fold (relative risk 2.39, 95% CI 2.18–2.61) and a 3-fold (relative risk 3.07, 95% CI 2.80–3.35) increased risk of IHD in men and women, respectively, in 2015. However, the absolute and relative differences in stroke decreased over time. For example, in the middle deprivation quintile, stroke rates decreased from 3.5-fold in 1991 to 2.5-fold in 2015 in men (relative risk 3.55, 95% CI 3.07–4.11 and relative risk 2.60, 95% CI 2.32–2.91, respectively) and women (relative risk 3.55, 95% CI 3.08–4.10 and relative risk 2.60, 95% CI 2.32–2.91, respectively; Table 3 and Supplementary Figure 4).

**Table 3:** Relative risks for ischaemic heart disease and stroke, among people aged 65 and 70 years, respectively, and in the middle deprivation quintile, comparing people with a hospital admission for each mental disorder versus no mental illness, by gender and year, based on predictive values from quasi-Poisson regression models

Year	Men Relative risk (95% CI)			Women Relative risk (95% CI)		
	Schizophrenia	Bipolar disorder	Depression	Schizophrenia	Bipolar disorder	Depression
Ischaemic heart disease						
1991	1.81 (1.49–2.20)	1.45 (1.26–1.68)	2.56 (2.26–2.90)	2.37 (1.95–2.87)	1.72 (1.50–1.97)	3.29 (2.91–3.72)
2000	1.53 (1.32–1.77)	1.57 (1.40–1.77)	2.59 (2.39–2.80)	2.00 (1.72–2.32)	1.86 (1.66–2.08)	3.32 (3.07–3.60)
2010	1.71 (1.50–1.95)	1.57 (1.40–1.75)	2.25 (2.10–2.41)	2.23 (1.95–2.56)	1.85 (1.66–2.07)	2.89 (2.69–3.10)
2015	1.91 (1.61–2.27)	1.60 (1.37–1.87)	2.39 (2.18–2.61)	2.50 (2.10–2.98)	1.89 (1.61–2.21)	3.07 (2.80–3.35)
Stroke						
1991	1.86 (1.42–2.43)	2.12 (1.80–2.51)	3.55 (3.07–4.11)	2.25 (1.74–2.90)	2.33 (1.99–2.73)	3.55 (3.08–4.10)
2000	2.20 (1.81–2.67)	1.94 (1.68–2.24)	3.24 (2.93–3.58)	2.66 (2.20–3.20)	2.13 (1.87–2.44)	3.24 (2.94–3.58)
2010	2.07 (1.73–2.48)	1.93 (1.68–2.23)	2.84 (2.59–3.11)	2.50 (2.09–2.98)	2.12 (1.86–2.42)	2.84 (2.60–3.11)
2015	1.87 (1.48–2.37)	1.80 (1.48–2.18)	2.60 (2.32–2.91)	2.26 (1.79–2.86)	1.97 (1.63–2.39)	2.60 (2.32–2.91)

## Discussion

We found that, in Scotland, mental health and IHD/stroke associations by time, age, gender and deprivation differed across three psychiatric disorders and by outcome. IHD and stroke incidence has generally declined over the past 25 years in all groups, but the relative disparity in risk between those with bipolar disorder or schizophrenia and those with no mental illness has remained stable. In contrast, the association between depression and stroke has slightly attenuated over time. However, the magnitude of effect of depression on IHD and stroke risk remains higher than for schizophrenia and bipolar disorder. Stroke and IHD incidence were higher in men than women in all groups, and age at event onset was considerably lower in people with psychiatric disorders compared with people with no record of hospital admission for mental illness. Among people with schizophrenia, higher socioeconomic status does not appear to mitigate the excess risk of stroke in some age groups. Being in the most deprived compared with the least deprived group conveys a greater absolute risk of IHD (but not stroke) for people with bipolar disorder or depression.

Our study has various strengths. To our knowledge, this is the largest study of people with bipolar disorder and risk of IHD and stroke to date, with more events than in all previous cohort studies combined.^11^ Similarly, it is one of the largest cohort studies on schizophrenia and IHD or stroke incidence.^10^ Furthermore, to the best of our knowledge, this is the first study to examine time trends in the incidence of IHD and stroke by psychiatric disorder status, and the first to examine differences by any indicator of socioeconomic status in a longitudinal study design. The use of national-level data meant that we included a large number of people with history of hospital admission for each mental disorder and a large number of outcomes, thus facilitating investigation of time trends and interactions with sociodemographic factors. In addition, IHD and stroke ascertainment was objective, with events ascertained via quality-assured hospital admission and national mortality records.^18^

Our study does have some shortcomings. The main limitation is that, in the absence of national primary care data for research purposes, our definition of psychiatric disorder was based on hospital admission records only, and so we are likely to have included people from the more severe end of the psychiatric disorder spectrum. Therefore, our findings may not necessarily be generalisable to people with these disorders who have never been admitted to hospital, although it is reasonable to assume that most individuals with schizophrenia or bipolar disorder will have had at least one hospital admission. This limitation is mitigated by the fact that hospital records extend back to 1981. Because we included people with a diagnosis of depression within acute hospital admission records, people with less severe depression may have been included because they were admitted to hospital for an unrelated disease or emergency. It is difficult to speculate how the definition of depression might have affected the results as bias may have occurred in both directions. A second limitation is that we will not have identified stroke or IHD events for which people were not admitted to hospital. We were also unable to adjust for factors known to be associated with psychiatric disorders and to be major risk factors for IHD and stroke, such as smoking, type 2 diabetes and body mass index, which could confound and/or mediate the associations. Similarly, prescription data were not available at a national level for the period of interest. Studies that have adjusted for lifestyle and disease history have found mixed results, but some indicate that an association persists even after adjusting for these factors.^9^ Very few studies have adjusted for psychotropic medication use. Finally, psychiatric hospital admission patterns in Scotland have changed over time, with reductions in psychiatric hospital admissions in favour of increased care in the community. Therefore, people included in the latter years of our study who were identified through psychiatric hospital admissions may differ from those who were admitted to hospital in the earlier period of the study.

Our finding that having a psychiatric disorder is associated with an increased risk of IHD and stroke concur with those of previous large cohort studies.^9^ However, our study is one of the first to convincingly show that bipolar disorder is associated with IHD risk, supporting findings from a previous study^19^ and making an important contribution to the relatively sparse literature in this area.^20^ A wide spectrum of factors are thought to explain the increased CVD risk in people with a psychiatric disorder, including lifestyle behaviours, increased risk of diabetes and side-effects of psychotropic medication.^20–22^ There may also be intrinsic physiological effects of the mental disorder itself; for example, there is accumulating evidence suggesting that glucose homeostasis is already impaired at schizophrenia onset.^23^ There may also be shared genetic links between psychiatric disorders and cardiometabolic disorders.^24,25^ Furthermore, people with psychiatric disorders may be underscreened^26,27^ and undertreated for key cardiometabolic risk factors,^28,29^ and receive suboptimal care for diabetes.^27,30^ The earlier onset of CVD in people with these disorders highlights the particular importance of primary prevention of cardiovascular disease in these groups, and suggests that cardiovascular risk factor screening should start at a younger age than in the general population. Men in general, and men and women from lower socioeconomic groups with a psychiatric disorder, are at particularly high risk of premature CVD. These sociodemographic patterns should be reflected in clinical guidelines to encourage better monitoring and minimise delay in the diagnosis and treatment of cardiovascular disease. However, despite universal healthcare provision in the UK, the inverse care law means that primary care resource allocation is not currently proportionate to need. General practices in more deprived areas have greater workloads, with a higher proportion of patients with complex needs, including comorbid physical and mental health problems, yet they do not receive additional funding.^31^ Additional resources therefore need to be appropriately allocated to support practitioners working in such areas to reduce inequalities in healthcare provision for people with complex health needs living in deprived areas.

To our knowledge, no other longitudinal study has reported on the associations between psychiatric disorders and risk of IHD or stroke by any indicator of socioeconomic status. Our finding that risk of IHD and stroke remains markedly elevated even in the least deprived groups highlights the negative impact that these psychiatric disorders have on physical health irrespective of area-based deprivation level. The variation in deprivation patterns observed across psychiatric disorders and cardiovascular outcomes could, however, indicate different underlying mechanisms between each mental disorder and each cardiovascular outcome and/or reflect differing patterns of access to and uptake of healthcare and primary prevention for cardiovascular disease. For example, stroke risk in people with schizophrenia did not decrease with decreasing deprivation in the same way that it did for IHD. This could indicate that the effect of schizophrenia on stroke risk is explained less by deprivation-related factors such as unhealthy lifestyle, and more by the intrinsic biological effects of schizophrenia or antipsychotic drug use. In contrast, living in a deprived area might exacerbate the risk of IHD in people with bipolar disorder and depression via higher prevalence of unfavourable lifestyle, such as smoking, physical inactivity and poor diet, and/or by less engagement with healthcare providers.

A previous Scottish study found no change in recent decades in the all-cause mortality gap between people with a hospital admission for mental illness and the general population.^32^ Our study indicates that, in addition, absolute but not relative rates of IHD and stroke incidence among those with schizophrenia and bipolar disorder have declined over time in this setting. Interestingly, we did find that relative rates of stroke (but not IHD) have declined somewhat among those with a hospital record for depression. Further investigation is needed to understand the reason for this, including the extent to which temporal changes in risk factors that are more strongly associated with stroke than IHD, such as blood pressure, contribute to this finding. We are unaware of any other published study on time trends of IHD or stroke incidence by psychiatric disorder status. In their systematic review, Correll *et al* found that, in meta-regression analyses, the magnitude of effect of psychiatric illness on CVD incidence was not greater in more recent compared with less recent studies, which concurs with our findings.^9^

Further studies on sociodemographic differences and temporal trends in the association between psychiatric illness and IHD and stroke incidence are needed to determine whether similar patterns are seen in other high-income settings. The reduction in IHD and stroke incidence over time among those with a hospital admission for psychiatric illness in Scotland is a welcome finding, but the persistent relative gap in incidence between those with and without psychiatric illness highlights the need for renewed efforts to reduce IHD and stroke risk. A multifaceted approach is required, given the complex underlying causes of excess CVD risk in these groups. However, there are still major gaps in our understanding of these mechanisms, particularly the role of psychotropic medication, that need to be addressed. Furthermore, the extent to which access to and/or uptake of appropriate cardiovascular screening, primary prevention and treatment of cardiovascular risk factors is optimal in people with psychiatric illness merits further investigation. Given the different sociodemographic patterns observed in our study, different monitoring, prevention and intervention approaches tailored to each psychiatric disorder and cardiovascular outcome may be required if we are to successfully reduce cardiovascular risk in this vulnerable population.

## Data Availability

The data that support the findings of this study are available from the Electronic Data Research and Innovation Service of National Health Service, National Services Scotland, but restrictions apply to the availability of these data, which were used under license for the current study, and so are not publicly available. Data are, however, available upon reasonable request and with permission of the Public Benefit and Privacy Panel of National Health Service, National Services Scotland.

## Supplementary material

For supplementary material accompanying this paper visit https://doi.org/10.1192/bjp.2019.250. click here to view supplementary material
