# Supplementary material for: Incidence of ischaemic heart disease and stroke among people with psychiatric disorders: retrospective cohort study
Source: Br J Psychiatry. 2020 Aug;217(2):442–9. doi: 10.1192/bjp.2019.250 (PMC7511900; doi:10.1192/bjp.2019.250)

# Cardiovascular disease incidence among people with major mental disorders in Scotland, 1991-2015: a retrospective cohort study

## Supplementary material

Supplementary Table 1 Mental disorder groups and associated ICD codes*

| Mental disorder | ICD10 codes | ICD9 codes |
|---|---|---|
| Mental and behavioural disorders due to psychoactive substance use | F10-F19 | 291.0-292.9; 303.0-305.8 |
| **Schizophrenia, schizotypal and delusional disorders** | F20-F29 | 295.0-295.9; 297.0-297.9; 298.1; 298.3; 298.4; 298.8; 298.9 |
| • Schizophrenia & schizoaffective disorders | F20 & F25 | 295.0-295.3; 295.6-295.9 |
| • Other psychoses (schizotypal disorders; acute & transient psychosis; delusional disorders and other psychotic disorders) | F21-F24; F28 & F29 | 295.4; 295.5; 297.0-297.9; 298.1; 298.3; 298.4; 298.8; 298.9 |
| **Mood (affective) disorders** | F30-F39 | 296.0-296.9; 298.0; 311; |
| • Bipolar disorder (manic episode & bipolar affective disorder) | F30-F31 | 296.0-296.1; 296.4-296.7 |
| • Depressive episode; recurrent depressive disorder; | F32-F33 | 296.2-296.3; 298.0; 311 |
| • persistent mood (affective) disorder; other mood (affective) disorders & unspecified mood (affective) disorders | F34, F38 & F39 | 296.8; 296.9 |
| **Neurotic, stress-related and somatoform disorders** | F40-F48 | 300.0-300.9; 298.2; 306.0-306.9; 307.8-309.0 |
| • Anxiety and obsessive compulsive disorders, stress-related & adjustment disorders and other neurotic disorders | F40-F43 & F48 | 300.0-300.9; 308.0-309.99 |
| • Dissociative disorders & somatoform disorders and other neurotic disorders | F44 & F45 | 298.2; 306.0-306.9; 307.8-307.89 |
| **Behavioural syndromes associated with physiological disturbances and physical factors** | F50-F59 | 307.1; 307.4-307.5; 316-316.9 |
| • Eating disorders | F50 | 307.1; 307.5 |
| • Non-organic sleep disorders & other behavioural syndromes associated with physiological disturbances and physical factors | F51-F59 | 307.4; 316-316.9 |
| Disorders of adult personality and behaviour | F60-F69 | 301.0-302.9 |
| Disorders of psychological development | F80-F89 | 299.0-299.9; 315.0-315.9 |
| Behavioural and emotional disorders with onset in childhood and adolescence | F90-F98 | 312.0-314.9; 307.0-307.3; 307.6-307.7 |
| Unspecified mental disorder | F99 | No equivalent code |

*headings in bold represent broad groups, which in this algorithm are further separated into subgroups for analyses of specific disorders

Supplementary Table 2 Age-standardised incidence of ischaemic heart disease and stroke among adults aged 40 years or over in Scotland, by mental health disorder, stratified by sex and time period, for 1990-2015

| | Age-standardised incidence rate, per 1000 person-years* (95% CI) | | | |
|---|---|---|---|---|
| Time period | No mental illness | Schizophrenia | Bipolar disorder | Depression |
| MEN | | | | |
| Ischaemic heart disease | | | | |
| 1991-1995 | 18.2 (18.1 - 18.4) | 26.1 (22.9 - 29.2) | 22.8 (20.6 - 25.1) | 37.2 (33.5 - 40.9) |
| 1996-2000 | 15.7 (15.5 - 15.8) | 21.9 (19.4 - 24.4) | 22.8 (20.5 – 25.0) | 37.0 (34.4 - 39.6) |
| 2001-2005 | 14.4 (14.3 - 14.6) | 18.0 (16.1 – 20.0) | 18.7 (16.7 - 20.7) | 27.4 (25.7 - 29.2) |
| 2006-2010 | 12.7 (12.6 - 12.8) | 16.0 (14.4 - 17.7) | 18.0 (16.1 – 20.0) | 24.3 (22.9 - 25.7) |
| 2011-2015 | 10.4 (10.3 - 10.5) | 14.7 (13.2 - 16.1) | 14.8 (13.1 - 16.4) | 19.4 (18.3 - 20.5) |
| Stroke | | | | |
| 1991-1995 | 7.2 (7.1 - 7.3) | 11.0 (8.7 - 13.3) | 13.2 (11.5 - 14.9) | 20.6 (17.9 - 23.2) |
| 1996-2000 | 6.2 (6.1 - 6.3) | 10.1 (8.3 - 11.8) | 8.5 (7.2 - 9.8) | 16.5 (14.8 - 18.3) |
| 2001-2005 | 5.4 (5.2 - 5.3) | 8.4 (7.0 - 9.9) | 8.1 (6.8 - 9.4) | 13.0 (11.8 - 14.2) |
| 2006-2010 | 4.35 (4.2 - 4.3) | 6.5 (5.4 - 7.6) | 5.6 (4.6 - 6.7) | 9.7 (8.8 - 10.6) |
| 2011-2015 | 3.7 (3.7 - 3.8) | 5.6 (4.6 - 6.5) | 5.0 (4.1 - 5.9) | 8.0 (7.3 - 8.7) |
| WOMEN | | | | |
| Ischaemic heart disease | | | | |
| 1991-1995 | 10.8 (10.7 - 10.9) | 16.9 (14.8 - 18.9) | 15.6 (14.5 - 16.7) | 27.4 (25.4 - 29.4) |
| 1996-2000 | 9.9 (9.8 - 9.9) | 13.1 (11.4 - 14.7) | 14.6 (13.6 - 15.7) | 24.3 (22.9 - 25.8) |
| 2001-2005 | 9.0 (8.9 - 9.0) | 13.2 (11.7 - 14.7) | 13.9 (12.9 - 15.0) | 21.3 (20.3 - 22.4) |
| 2006-2010 | 7.8 (7.7 - 7.8) | 12.9 (11.5) - 14.3) | 11.9 (10.9 – 13.0) | 17.0 (16.1 - 17.8) |
| 2011-2015 | 5.9 (5.8 – 6.0) | 11.5 (10.3 – 12.8) | 9.3 (8.4 - 10.2) | 14.0 (13.3 - 14.7) |
| Stroke | | | | |
| 1991-1995 | 5.7 (5.6 - 5.8) | 8.5 (7.0 - 10.0) | 9.7 (8.7 - 10.6) | 15.0 (13.6 - 16.4) |
| 1996-2000 | 5.1 (5.0 - 5.1) | 9.3 (7.9 - 10.7) | 7.7 (7.0 - 8.5) | 12.1 (11.2 - 13.1) |
| 2001-2005 | 4.4 (4.4 - 4.5) | 8.1 (6.9 - 9.3) | 7.5 (6.7 - 8.2) | 10.7 (10.0 - 11.5) |
| 2006-2010 | 3.6 (3.6 – 3.7) | 7.0 (6.0 - 8.1) | 5.9 (5.2 - 6.6) | 8.0 (7.5 - 8.6) |
| 2011-2015 | 3.2 (3.1 – 3.2) | 5.1 (4.3 – 6.0) | 4.9 (4.3 - 5.5) | 6.5 (6.1 – 7.0) |

*Standardised to the European Standard Population
CI = confidence interval

Supplementary Figure 1 Incidence (per 1000 person-years)* of ischaemic heart disease and stroke with increasing age, by mental health condition and sex, among people in the middle deprivation quintile, in Scotland in 2015

*Predicted incidence rates obtained from quasi-Poisson regression models including mental health status, age, sex, deprivation and time period, plus all mental health status and age interactions

IHD = ischaemic heart disease

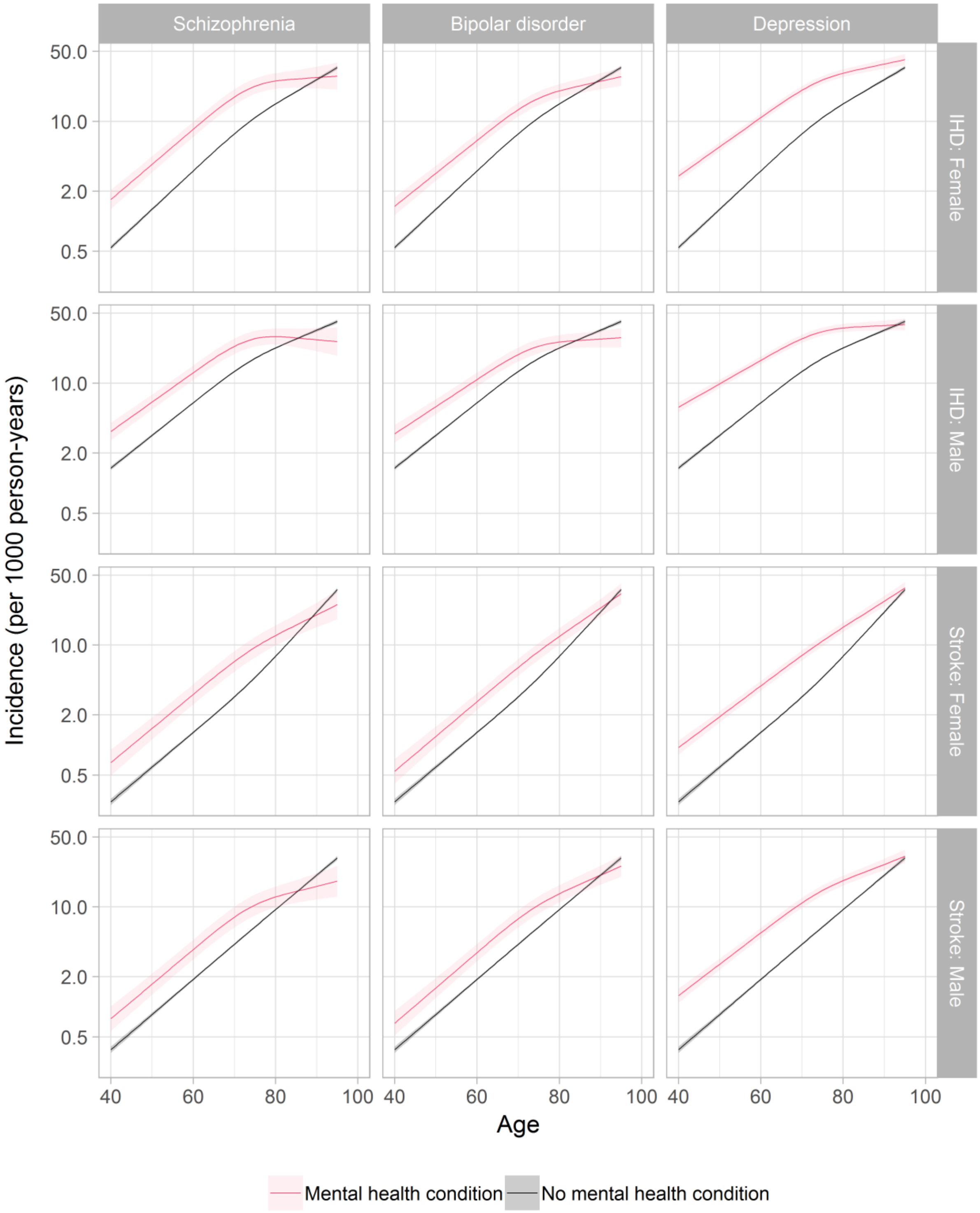

Supplementary Figure 2 Incidence (per 1000 person-years)* of ischaemic heart disease (IHD) and stroke by area-based deprivation level, for each mental health condition and by sex, among people aged 65 (IHD) and 70 (stroke) years, in Scotland in 2015

*Predicted incidence rates obtained from quasi-Poisson regression models including mental health status, age, sex, deprivation and time period, plus all mental health status and age interactions

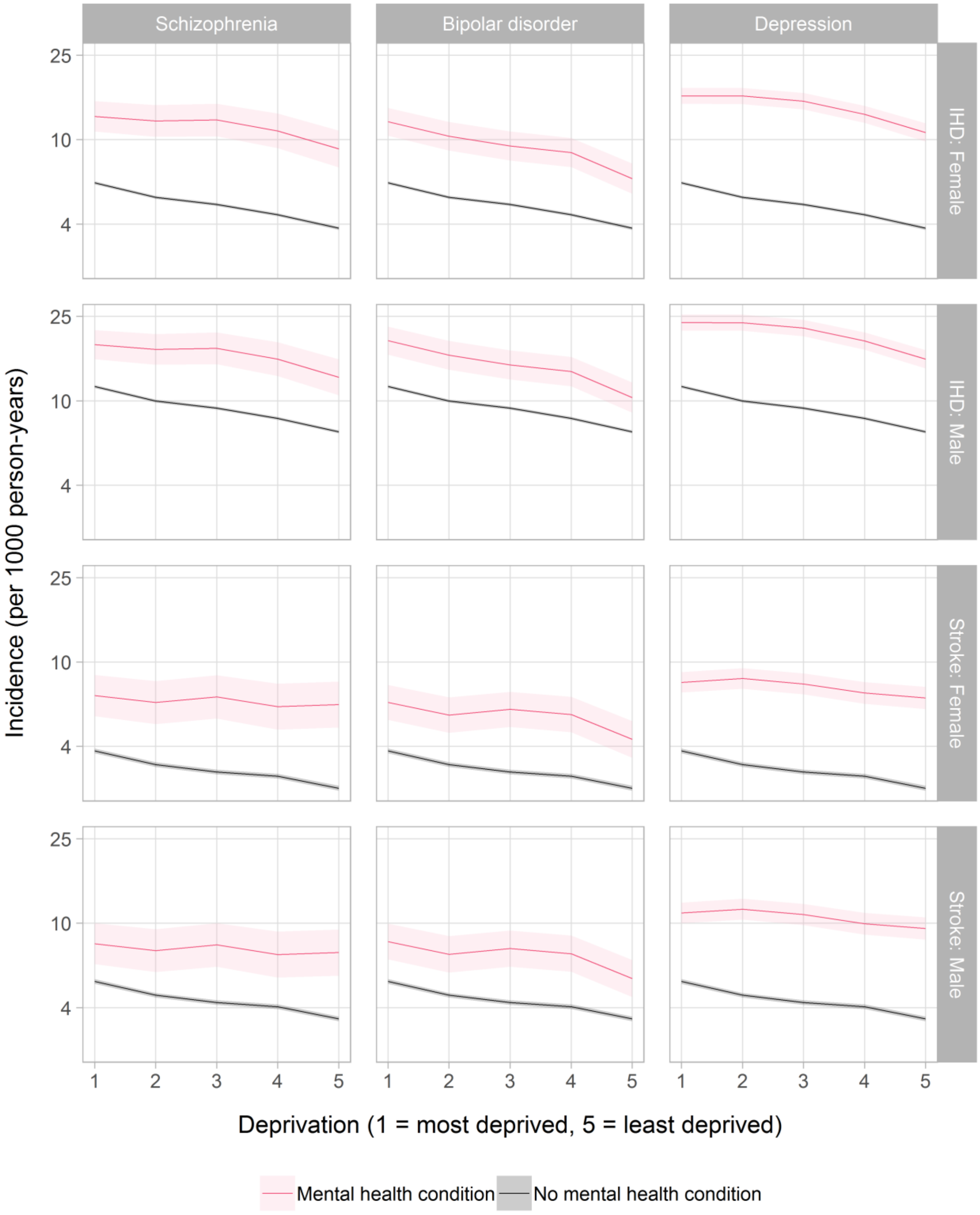

Supplementary Figure 3 Difference in absolute risk of (a) ischaemic heart disease (in people aged 65) and (b) stroke (in people aged 70), comparing each mental health disorder against the no mental illness group, for men and women, by deprivation in Scotland, 2015

(a)

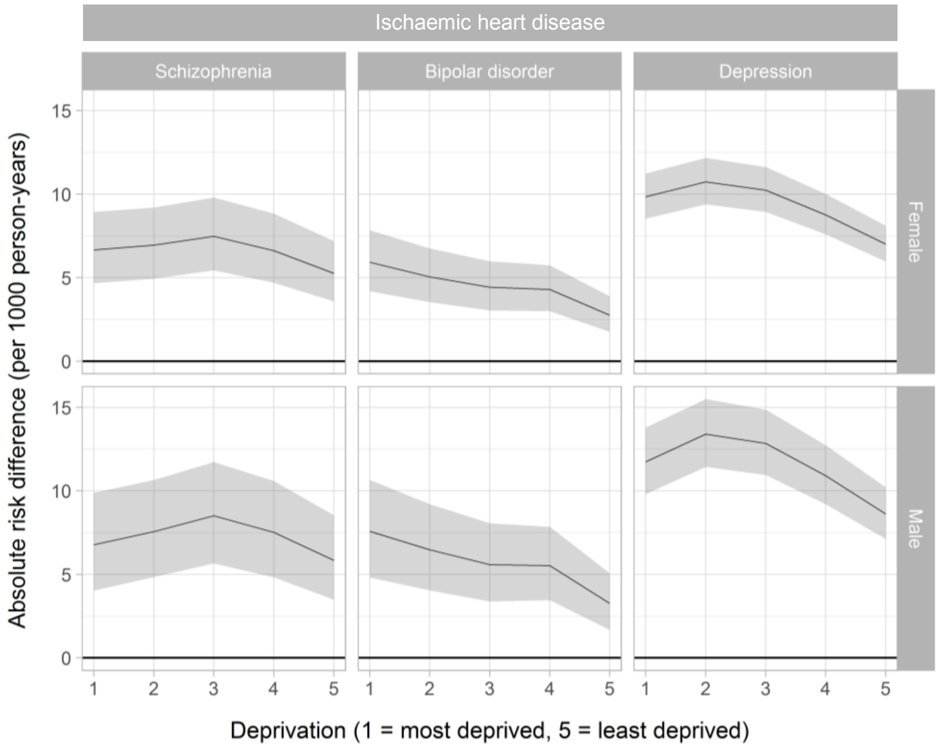


(b)

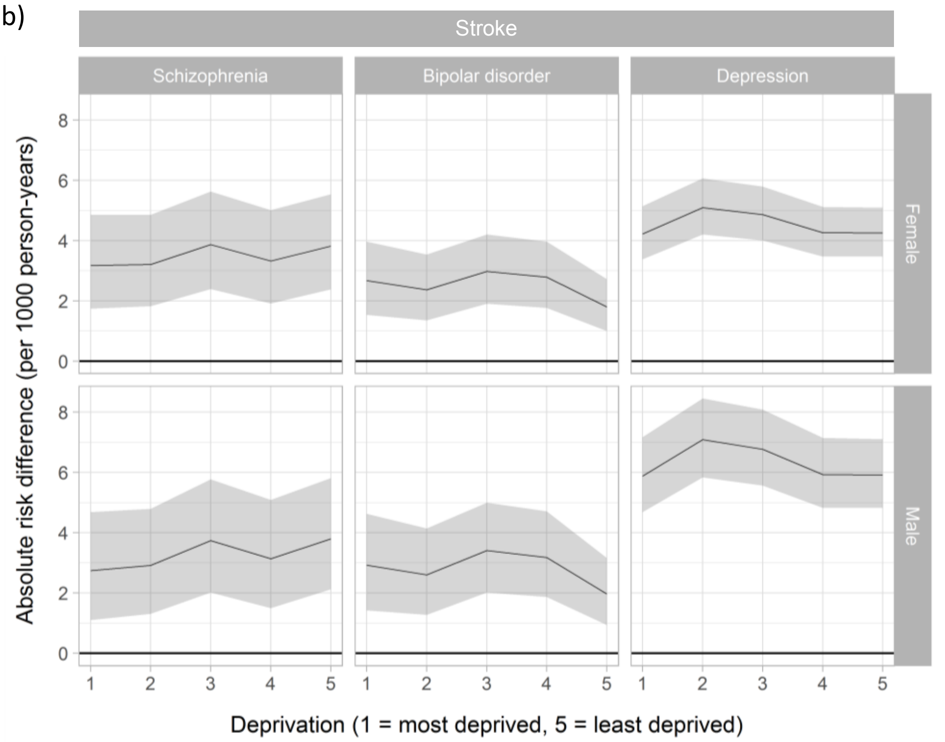

Supplementary Figure 4 Relative rates (with 95% confidence intervals)* of ischaemic heart disease (IHD) and stroke, comparing people with a history of hospitalisation for each mental disorder versus no mental illness by year, among those aged 65 (IHD) and 70 (stroke) years and in the middle deprivation quintile, in Scotland, from 1991-2015

*Predicted relative rates obtained from quasi-Poisson regression models including mental health status, age, sex, deprivation and time period, plus all mental health status and age interactions

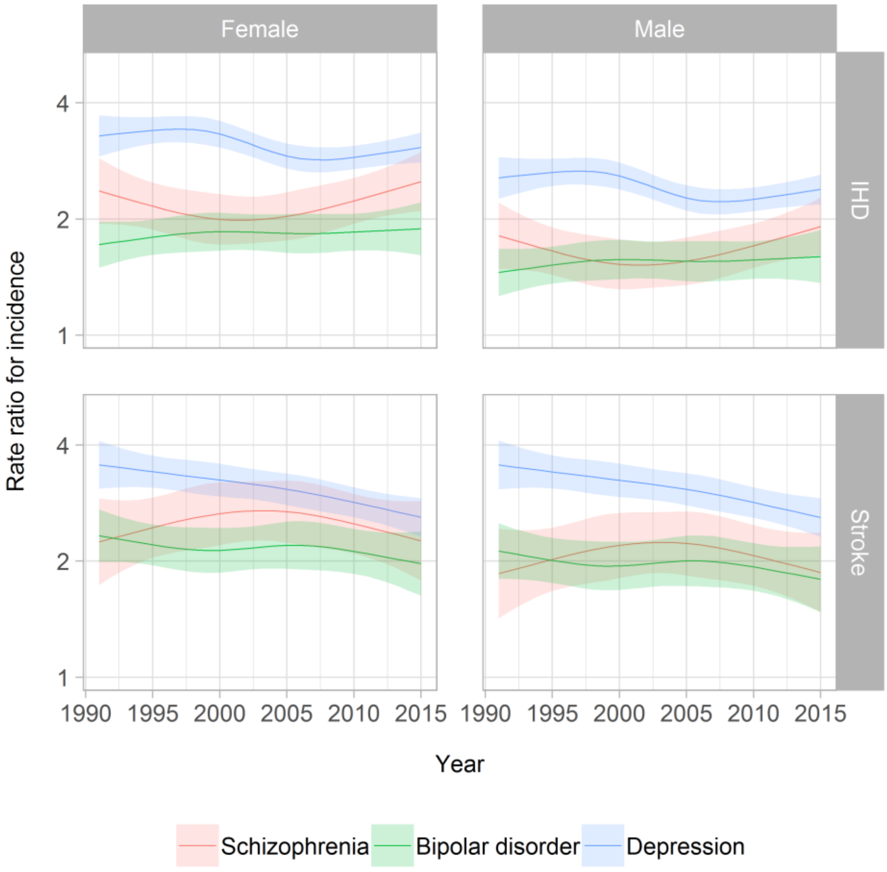

Supplement: Supplementary file 1 [file S0007125019002502sup001.pdf]
